# Potential Role of ACE Polymorphisms in Severe Malaria: A Case Report

**DOI:** 10.1155/crdi/9821533

**Published:** 2026-05-06

**Authors:** Raffaella Pisapia, Andrea Pomicino, Giuseppina Minei, Isabella Iadevito, Alessandro Perrella

**Affiliations:** ^1^ Emerging Infectious and Highly Infectious Diseases Unit, P.O. D. Cotugno - AORN Ospedali Dei Colli, Via Leonardo Bianchi snc, Naples, 80131, Italy; ^2^ Radiology Unit, P.O. D. Cotugno - AORN Ospedali Dei Colli, Via Leonardo Bianchi snc, Naples, 80131, Italy; ^3^ Regional Observatory for Infectious Diseases, Campania Region, Naples, Italy

**Keywords:** ACE polymorphisms, genetic studies, malaria, severity

## Abstract

Malaria, primarily caused by *Plasmodium falciparum*, remains a critical global health challenge, particularly in sub‐Saharan Africa. Genetic factors such as angiotensin‐converting enzyme (ACE) I/D polymorphism have been implicated in influencing malaria severity and outcomes. We report the case of a 40‐year‐old woman from Seychelles, residing in Ivory Coast, who developed severe *P. falciparum* malaria complicated by thrombosis. Laboratory findings revealed hyperparasitemia, acute kidney injury, and metabolic acidosis. Genetic testing through real time PCR identified the ACE I/D polymorphism, along with other thrombophilic variants such as MTHFR C677T and A1298C, suggesting a potential role in clinical course.

This case aims to contribute to the debate about the role of ACE polymorphisms in malaria severity. Comprehensive studies are warranted to explore the genetic landscape in malaria‐endemic regions and its implications for personalized treatment strategies.

## 1. Introduction

Malaria remains a critical global health challenge, with approximately 249 million clinical cases and 608,000 deaths reported annually. *Plasmodium falciparum* is the primary cause of malaria‐related morbidity and mortality, particularly due to its propensity to cause severe and life‐threatening disease manifestations [1]. The pathogenic mechanisms of severe malaria are complex and not fully elucidated. They likely involve intricate host‐parasite interactions, including cytoadherence and sequestration of parasitized erythrocytes in the microvasculature of critical organs, alongside inflammation and dysregulation of hemostatic processes [2, 3]. Cytokine gene polymorphisms have been reported to affect cytokine production, thereby disrupting the balance between pro‐inflammatory and anti‐inflammatory responses. This disruption may influence susceptibility to malaria and modulate disease progression [4]. Among these, the angiotensin‐converting enzyme (ACE) gene polymorphism (I/D variant) has been implicated. According to some evidences, the ‘D’ allele is associated with elevated ACE levels and increased angiotensin II (Ang II) production, which has shown potential protective effects against severe malaria [5]. The ACE I/D polymorphism consists of either the insertion (I allele) or deletion (D allele) of a 287‐base pair Alu repetitive sequence located in intron 16 of the ACE gene. The I allele corresponds to the presence of this 287‐bp fragment, whereas the D allele reflects its absence. This polymorphism influences circulating and tissue ACE levels, with the D allele and particularly the DD genotype being associated with higher ACE activity and increased angiotensin II production. There is still limited evidence about the role of ACE polymorphisms in the clinical course of *P. falciparum* malaria.

We report a case of severe malaria complicated also by venous thrombosis, focusing on the presence of the ACE I/D polymorphism and discussing its potential role in disease progression.

## 2. Case Description

A 40‐year‐old woman was admitted to our unit in August 2024 with a diagnosis of severe *Plasmodium falciparum* malaria. The patient, originally from Seychelles, had resided in Ivory Coast for 2 years before traveling to Italy for vacation with her family. The patient had no prior history of malaria but reported a previous episode of umbilical vein thrombosis during pregnancy. Upon admission, she exhibited fever (39°C), severe prostration, vomiting, and diarrhea. No neurological signs were present. Laboratory findings confirmed severe malaria with hyperparasitemia (> 10%), acute kidney injury (creatinine 3.4 mg/dL), hyperbilirubinemia (10.4 mg/dL), and metabolic acidosis (bicarbonates < 15 mmol/L). Additional abnormalities included thrombocytopenia (16,000/mm^3^), hepatic dysfunction (AST 472 U/L, ALT 188 U/L), and electrolyte imbalances (Table 1). Antimalarial therapy with intravenous artesunate (2.4 mg/kg at time 0, 12, 24, 48, and 72 h) was initiated. For the severe clinical condition and inability to take oral drugs, the intravenous artesunate course was prolonged, and clindamycin (10 mg/kg on Day 1, then 5 mg/kg three times a day) was initiated after 24 h. After 72 h of intravenous treatment, a 3‐day course of oral dihydroartemisinin‐piperaquine was administered. Parasite clearance was achieved by day seven. Cerebral involvement was excluded according to WHO criteria, based on preserved consciousness (GCS 15), absence of seizures or focal neurological deficits, and stable neurological status throughout hospitalization and follow‐up.

**TABLE 1:** Timeline of biochemical tests and clinical course.

Laboratory investigation (normal value)	Diagnosis of severe malaria		Pneumonia and subclavian vein thrombosis		Anemia		Recovery and discharge		
	Day 1	Day 3	Day 5	Day 7	Day 10	Day 14	Day 17	Day 20	Day 26
WBC (4.5–11 × 10^3^/μL)	6.67	8.95	15.0	15.0	12.4	5.46	5.27	4.49	4.14
RBC (3.5–5.5 × 10^6^/μL)	4.16	4.30	3.85	3.11	2.98	2.20	2.49	3.0	3.10
HB (12.0–16.0 g/L)	11,8	12.3	10.8	8.6	8.1	7.0	7.4	8.8	9.1
PLT (150–400 × 10^3^/μL)	16	19	21	147	198	247	232	175	188
Creatinine (0.55–1.04 mg/dL)	3.4	3.0	2.4	1.2	0.7	NP	0.4	0.5	0.4
Sodium (135‐145 mEq/L)	129	126	127	131	133	NP	138	140	136
Potassium (3.5–5.1 mEq/L)	4.4	3,4	3,3	3.3	3.9	NP	4.0	4.5	4.58
Calcium (8.7–10.4 mg/dL)	7,7	6.5	6.1	7.4	6.9	NP	8.2	9.5	9.9
AST (0–34 U/L)	472	295	230	68	38	NP	43	33	38
ALT (10–49 U/L)	188	147	138	85	52	NP	21	22	33
Bilirubin (0.2–1 mg/dL)	10.4	3.8	4.2	1.5	0.9	NP	1.7	1.6	1.0
INR (0.8–1.2)	1.15	1.00	0.95	1.34	1.30	NP	1.11	1.06	1.0
D‐Dimer (< 500 ng/dL)	114.360	27,897	9487	4693	4403	NP	7544	2627	1288
Plasma bicarbonate (22–24 mmol/L)	13.5	15	18.7	22.8	22.5	24.2	24.7	24.6	24.4
Parasitemia	> 10%	7.75%	0.2%	Negative	Negative	NP	NP	NP	NP

Abbreviation: NP, not performed.

**FIGURE 1:**
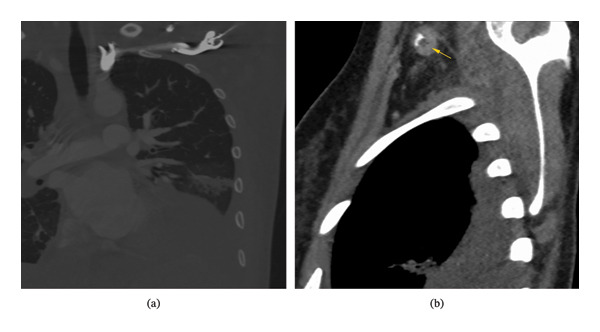
(a, b) Angio TC‐scan showing a proximal subclavian vein thrombosis.

The clinical course was complicated by bacterial pneumonia due to *H. influenzae*, already present at admission, and afterwards by a nosocomial pneumonia due to *S. aureus* and *P. aeruginosa* co‐infection, requiring oxygen therapy and antibiotic treatment with meropenem and linezolid. On Day 14, the patient developed anemia (hemoglobin 7.0 g/dL), necessitating a transfusion of one unit of packed red blood cells. A peripherally inserted central catheter (PICC) placed in the left arm on day one of admission led to thrombophlebitis and proximal subclavian vein thrombosis (Figures 1(a), 1(b)). Given her history of umbilical vein thrombosis, thrombophilia genetic testing through real time PCR was performed, revealing genetic variations at increased risk of thrombosis, including the presence of the ACE I/D genotype, consistent with an intermediate expression profile of ACE activity. Moreover, the patient was also tested heterozygous for MTHFR C677T and A1298C and positive for HPAI T1565C GpIIIa, all of which are noted as increasing the risk of thrombosis (Table 2). Tests for antiphospholipid antibodies, homocysteine levels, and autoimmune markers (ANA and anti‐dsDNA) were negative. The patient was discharged in good clinical condition after 26 days, with complete recovery.

**TABLE 2:** Thrombophilia genetic testing.

Mutation	Genotype	Phenotype
FV Leiden G1691A/FVH1299R	Wild type	No increase in risk of thrombosis
FIIG20210A	Wild type	No increase in risk of thrombosis
MTHFR C677T	Heterozygous	Increased risk of vascular disease
MTHFR A1298C	Heterozygous	Increased risk of vascular disease
CBS 844ins68 (I/D)	D	No increase in risk of thrombosis
PAI‐1–675	4 G/5G	Increased risk of type II diabetes
HPAI T1565C GpIIIa (L33P)	T/C	Increased risk of thrombosis and cardiovascular disease
ACE	I/D	Increased risk of thrombosis
ApoE T112C and T158C	e4/e4	Increased risk of atherosclerosis
AGT (g.T9543C)	T/C	Increased risk of cardiovascular disease
ATR‐1 (A1166C)	A/C	Increased risk of myocardial infarction
FGB‐455 G > A	G/G	No increase in risk of thrombosis
FXIII (g.G7130T)	G/G	No increase in risk of thrombosis

In order to describe this case, a detailed review of the electronic medical records was performed to collect clinical, laboratory, and imaging data related to the patient’s course. Clinical management was carried out as part of routine care.

## 3. Discussion

*Plasmodium falciparum* is the leading cause of severe malaria globally, with pathogenic mechanisms involving cytoadherence, microvascular sequestration, and excessive inflammatory responses [7, 8]. Polymorphisms in genes regulating immune mediators and inflammatory cytokines can significantly influence the course and severity of the disease [4, 9]. The Renin‐Angiotensin System (RAS) plays a crucial role in influencing the course of both non‐communicable and infectious diseases. ACE and ACE2 are central enzymes of the RAS ACE converts angiotensin I to Ang II, a vasoconstrictor, while ACE2 converts Ang II to angiotensin (1–7), a vasodilator [10–13]. The polymorphism of the ACE gene is characterized by the absence (D allele) or the presence (I allele) of a 287 base pair Alu repeat in intron 16. Variations in the ACE I/D polymorphism have been associated with altered enzyme levels, influencing disease outcomes in conditions such as hypertension, diabetes, COVID‐19, and malaria [10, 14, 15]. Evidence suggests that elevated Ang II levels in the DD genotype might have protective effects against malaria, including reduction of cerebral malaria rates. For instance, Ang II and related peptides have demonstrated antiplasmodial activity in vitro and in vivo [14]. Additionally, studies in Indian and Nigerian populations have linked the ACE I/D polymorphism to malaria severity, including cerebral malaria, suggesting that the D/D genotype confers a protective effect [15, 16]. Similarly, research in Ghana has hypothesized that the high frequency of the D/D genotype may reflect evolutionary selection against severe malaria [17].

In the present case, the ACE I/D polymorphism, along with other heterozygous variants predisposing for thrombosis [18], likely contributed to the patient’s thrombophilic predisposition and might have influenced the disease course. This highlights the multifaceted interplay between host genetic factors and malaria pathogenesis. However, as this is a single case report, the findings should be interpreted with caution. Large‐scale studies are needed to further explore the role of genetic polymorphisms in malaria. Such research could pave the way for novel therapeutic approaches targeting host‐pathogen interactions and improving disease management. The high frequency of the ACE D allele in African populations highlights its importance in malaria‐endemic regions. Understanding its role could inform risk stratification and targeted interventions. Thrombosis in malaria, as seen here, underscores the need for genetic screening in severe cases to guide management.

## 4. Conclusions

The high frequency of the ACE D allele in African populations highlights its importance in malaria‐endemic regions. Understanding its role could inform risk stratification and targeted interventions. Thrombosis in malaria, as seen here, underscores the need for genetic screening in severe cases to guide management. Particularly the multifactorial pathogenesis of severe malaria, where genetic predispositions like ACE I/D polymorphism may influence disease severity and complications. The high prevalence of the D allele in African populations suggests a potential evolutionary advantage, but its role in thrombotic events requires further exploration. Comprehensive genetic studies in malaria‐endemic regions are crucial to elucidate the impact of ACE polymorphisms and other genetic factors. Such insights could pave the way for personalized treatment strategies, improving outcomes in high‐risk populations [6].

## Funding

No specific funding has been used for the drafting of this manuscript.

## Consent

As this report describes standard clinical care with no experimental procedures, ethical committee approval was not required. Written informed consent for publication was obtained from the patient.

## Conflicts of Interest

The authors declare no conflicts of interest.

## Data Availability

Data are available on request due to privacy/ethical restrictions.
